# Development of novel bioadhesive granisetron hydrochloride spanlastic gel and insert for brain targeting and study their effects on rats

**DOI:** 10.1080/10717544.2017.1413447

**Published:** 2017-12-12

**Authors:** Rehab Abdelmonem, Mohamed el Nabarawi, Alshaimaa Attia

**Affiliations:** ^a^ Department of Pharmaceutics and Industrial Pharmacy, Faculty of Pharmacy, Misr University for Science and Technology 6th of October City, Giza, Egypt; ^b^ Department of Pharmaceutics and Industrial Pharmacy, Faculty of Pharmacy, Cairo University Giza Egypt

**Keywords:** Granisetron hydrochloride, spanlastic dispersions, mucoadhesive gels, lyophilized inserts, intranasal brain targeting

## Abstract

The aim of this study was to formulate granisetron hydrochloride (GH) spanlastic in mucoadhesive gels and lyophilized inserts for intranasal administration to improve GH bioavailability and brain targeting. Carpapol 934 and HPMC were incorporated in GH spanlastic in nasal gels (GHSpNGs). Gelatin and HPMC as matrix former, glycine as a collapse protecting and mannitol as an insert filler and sweeting agent were used to prepare GH spanlastic loaded in lyophilized inserts (GHSpNIs). The prepared GHSpNGs were characterized for pH measurement, drug content, rheology, and *in vitro* drug release. The prepared GHSpNIs were characterized for drug content, surface pH, GH release, and mucoadhesion. Biological investigations including pharmacokinetics studies and brain drug targeting efficiency dimensions were performed on rats (LC–MS/MS). The results showed thixotropic pseudoplastic gels and white insert with pH values in a physiological range, drug content (89.9–98.6%), (82.4–98.38%) for gel and insert, respectively and rapid release rate of GH. Biological studies showed that *C*_max_ and AUC_0–6 h_ in brain and plasma after intranasal administration of gel and insert were higher compared to IV administration of GH solution. A high brain targeting efficiency (199.3%, 230%) for gel and insert, respectively and a direct nose to brain transport (49.8%, 56.95%) for gel and insert, respectively confirmed that there is a direct nose to brain transport of GH following nasal administration of GH spanlastic loaded in nasal gel and insert. GHSpNIs can be considered as potential novel drug delivery system intended for brain targeting via the nasal rout of administration than GHSpNGs.

## 1. Introduction

Site to specific targeted drug delivery systems aimed at localizing drugs at their desired site of action, reducing toxicity, and increasing treatment efficiency. In spite of its relatively high blood flow, brain targeting remains one of the most challenging research areas in pharmaceutical science due to efficient brain protection (Bisht, 2011). Among the major obstacles are the blood–brain barrier (BBB) and the blood–cerebrospinal fluid barrier (BCSFB) (Alam et al., 2010). The former is a semipermeable selective membrane that does not only function as a physical barrier, but also as a biochemical barrier expressing certain enzymes and efflux p-glycoprotein (Hawkins & Davis, 2005). Thus, the BBB is often the rate-limiting factor in determining permeation of therapeutic drugs into the brain. With the help of BCSFB and meninges; it also controls the brain internal environment. Strategies relying on manipulating BBB, by passing it or using carrier system can be used for drug delivery to the brain (Gabal et al., 2014). Intranasal route is one of these strategies for drug delivery to the brain through three different pathways: systemically where the drug crosses the BBB, through the olfactory region and the trigeminal pathway where it is transported directly from nasal cavity to the central nervous system (Kumar et al., 2008). Spanlastic dispersions can be designed to entrap hydrophilic therapeutics like GH, where the transport across the BBB to reach the brain is based on the characteristics of spanlastic dispersion and not on that of the therapeutic agent (Alexis et al., 2008). Granisetron is a potent, highly selective antagonist of 5-HT3 receptors. The antiemetic activity of the drug is brought about through the inhibition of 5-HT3 receptors present both centrally (medullary chemoreceptor zone) and peripherally (GI tract). Granisetron is used in the management of nausea and vomiting associated with emetogenic cancer chemotherapy, including high dose cisplatin and radiotherapy. It is used also for the prevention and treatment of postoperative nausea and vomiting (Ahmed et al., 2014).

In the market, granisetron is available in the form of tablets and injection. Those currently marketed products each have advantages and disadvantages. The intravenous dose, with 100% bioavailability, suffers from patient compliance, while the oral tablet, which is relatively easier in administration, is extensively metabolized in the liver. Therefore, granisetron delivery *via* intranasal route may avoid drug inactivation in the liver.

## 2. Materials and methods

### 2.1. Materials

Granistron hydrochloride (GH) was kindly provided by Amoun Pharmaceuticals, Egypt. Span 60, tween 60, and tween 80 were purchased from Sigma Chemical Co, St. Louis, MO. Triethanolamine, Carbopol 934, and hydroxypropyl methylcellulose (HPMC) B.F., Goodrich Chemical Company (PV, CA), OH, and Tama, Tokyo, Japan, respectively. Gelatin, El-Nasr Company for Pharmaceuticals, Cairo. Mannitol and agar were purchased from AL Shark Alawsat Pharmaceutical Chemical Company, Cairo, Egypt. Potassium dihydrogen phosphate, disodium hydrogen phosphate, and xylene were purcashed from El-Nasr Pharmaceutical Co. for Chemicals Cairo, Egypt. Acetonitrile, formic acid, methanol, ethanol and tertiary butyl methyl ether (TBME), HPLC grade, and toresemide (internal standard for LC-MS/MS) were acquired from Sigma Aldrich (St. Louis, Missouri). All other chemicals were of analytical grade and used as received.

### 2.2. Methods

DSC study was performed. The samples (3–4 mg) were heated in hermetically sealed flat bottomed aluminum pans over a temperature range of 30–200 °C, at a constant heating rate of 10 °C/min, under an inert nitrogen flow of 30 ml/min.

#### 2.2.1. Formulation and evaluation of GH-spanlastic dispersions

GH-loaded spanlastic dispersions were prepared by the ethanol injection method. Briefly, GH and Span^®^ 60 were dissolved in ethanol and injected into a preheated aqueous phase in which an edge activator (Tween^®^ 60 or Tween^®^ 80) was previously dissolved. The organic phase to the aqueous phase ratio was fixed as 1:5. The investigated Span^®^:edge activator ratios were 100:0, 90:10, 80:20, 70:30, 60:40, and 50:50, respectively. The spanlastic vesicles were formed spontaneously and turned the resulting hydroalcoholic solution slightly turbid. Continuous stirring of the latter solution on a magnetic stirrer was performed to allow complete evaporation of ethanol and subsequent formation of GH-loaded aqueous spanlastic dispersions. To promote the development of fine spanlastic dispersions, ultrasonic water-bath sonication (Crest Ultrasonics Corp., Trenton, NJ) was performed for 5 min. The prepared spanlastic dispersions were evaluated for particle size, zeta potential, entrapment efficiency *in vitro* drug release, and morphological examination *via* TEM. The results were shown in our previous paper (Tayel et al., 2015; El Nabarawi et al., 2016).

#### 2.2.2. Formulation of SpG containing the spanlastic dispersions

The selected spanlastics formulae S4 and S9 were chosen according to the particle size (200–500), high entrapment, PI <1, and negative zeta (high stable). Two polymers were used, HPMC and Cabopol (Table 1). Gels were prepared by gradually adding the calculated amount of HPMC (2% and 4%), while stirring to 1/3 of the required amount of distilled water at 80 °C (Hwang et al., 1999). The final volume was then adjusted to 100 ml by distilled water containing GH-loaded spanlastic dispersions. The prepared gels were stored overnight in the refrigerator. Carbopol 934 (1% and 2%) powder was sprinkled gradually into a vortex of 100 ml of distilled water containing the selected spanlastic dispersions S4 (10 mg GH, 140 mg span 60, 60 mg tween 80), or S9 (10 mg GH, 140 mg span 60, 60 mg tween 60) (El Nabarawi et al., 2016) with an equivalent amount to 2 mg GH/gm gel in 250 ml beaker, and stirred with a mechanical stirrer at a high speed until a thin dispersion without lumps was obtained. The stirring speed was then reduced in order to break the foam. Few drops of triethanolamine were then added at once to form the base (Koleng et al., 2006).

**Table 1.** The composition of GSpNG formulae.

Formula	Spanlastic dispersions	HPMC (%w/w)	Carbopol (%w/w)
G1	The chosen Spanlastic dispersions S4 (10 mg GH, 140 mg span 60, 60 mg tween 80) S9 (10 mg GH, 140 mg span 60, 60 mg tween 60)	2%	–
G2		2%	–
G3		4%	–
G4		4%	–
G5		–	1%
G6		–	1%
G7		–	2%
G8		–	2%

##### 2.2.2.1. *In vitro* evaluation of GH spanlastic nasal gelformulae (GHSpNGs)

###### 2.2.2.1.1. Visual inspection

The appearance and other physical properties, including color, precipitation, and homogeneity of freshly prepared gels were inspected by visual inspection under black and white background.

###### 2.2.2.1.2. pH measurement

The pH of various GHSpNGs was determined by using a digital pH meter. One gram of gel was dissolved in 9 ml of distilled water using a magnetic stirrer, warming over water bath if needed. Subsequently, the pH was measured after cooling to room temperature and storing for 2 h. The measurement of the pH of each formula was done in triplicate and the average values calculated.

###### 2.2.2.1.3. Determination of drug content

One gram of the prepared gel was mixed with 100 ml ethanol using an Incubator Shaker. Aliquots were withdrawn and diluted, after filtering the solution, and the absorbance was measured at *λ*(max) 301.60 nm. This procedure was done in triplicate manner for each formula. The average drug content and standard deviation were calculated for each formula.

###### 2.2.2.1.4. Spreadability of GHSpNGs

It is determined by using two glass slides, the lower one is fixed and the upper one is movable. Half gram of the formulated GHSpNG was placed between the fixed slide and the upper movable slide. A fixed weight was placed above the upper slide. The distance diameter traveled by gel was determined. Measurements were repeated three times for each of the gel preparation (Shivhare et al., 2009).

###### 2.2.2.1.5. Rheology study of all GHSpNGs

The viscosity of different bases prepared with different concentrations was determined using cone and plate viscometer (Brookfield Viscometer model III). The freshly prepared gels were placed in the cup of the viscometer using spindle 52 at 25 °C ± 1.

###### 2.2.2.1.6. *In vitro* GH release from different nasal gel formulae

The study was carried out using a modified USP dissolution apparatus II. A 10 ml capacity syringe was prepared to act as a tube by cutting smoothly the whole diameter near the nozzle (El-Hadidy, 2010). Accurately weighed one gram of each gel formulae was introduced into the syringe from the top after removing the pump. The syringe was covered upside with plastic cover and then attached to the rotating paddle. The syringe tube was immersed in the vessel containing 200 ml distilled water at 37 °C ± 0.5 with a paddle speed of 50 rpm. Aliquot of (5 ml) was withdrawn at specified time interval over 3 h and immediately replaced with fresh release medium. The drug content in the withdrawn samples was determined spectrophotometrically at 301.60 nm (UV spectrophotometer; UV-1650 P.C Shimadzu, Japan). The results are the mean and standard deviation values of three runs.

###### 2.2.2.1.7. Determination of mucodhesion of GHSpNG formulae

The experimental technique used for determining the bioadhesive force has been derived from a previously published method (Shivhare et al., 2009). The apparatus was designed for measuring the minimum weight required for detachment of two membranes from each other with a film of polymer spread between them as described in Yong et al. (2001) and El-Nabarawi et al. (2012).

#### 2.2.3. Formulation of GH spanlastics nasal inserts (GHSpNI)

According to TEM micrographs results of the prepared GH spanlastics (El Nabarawi et al., 2016), eight nasal inserts were prepared by adding gelatin (1% w/w) or HPMC (2% w/w) as matrix former, glycine (0.5% and 1% w/v) as collapse protecting and 20 gm mannitol as insert filler and sweeting agent (to avoid bitter taste of postnasal secretion) to one-third of required amount of distilled water (Table 2). The selected (S4, S9) spanlastic dispersion (El Nabarawi et al., 2016) with an equivalent amount 2 mg GH/ml was added to the aqueous solution containing the matrix former, mannitol, and glycine using a magnetic stirrer. The resultant solution was made up to 100 ml by distilled water and stirred to homogeneity. One milliliter of the suspension was then poured into the polypropylene tubes as mold. The tubes were then transferred to a freezer at −22 °C and kept for 24 hs. Frozen inserts were then placed for 24 h in a freeze-dryer. The lyophilized inserts were not sticky to the tubes due to shrinkage occurred then removed and kept in tightly closed containers in desiccators over anhydrous calcium chloride at room temperature (Sherimeier & Schmidt, 2002).

**Table 2.** The composition of the GHSpNIs.

Formula	Spanlastic dispersions	HPMC (%w/w)	Gelatin (%w/w)	Glycine (%w/w)	Mannitol (%w/w)
F1	The best chosen dispersionS4 (10 mg GH, 140 mg span 60,60 mg tween 80)S9 (10 mg GH, 140 mg span 60, 60 mg tween 60)	2%	–	0.5%	20
F2		2%	–	0.5%	20
F3		2%	–	1%	20
F4		2%	–	1%	20
F5		–	1%	0.5%	20
F6		–	1%	0.5%	20
F7		–	1%	1%	20
F8		–	1%	1%	20

##### 2.2.3.1. Evaluation of the prepared nasal inserts

###### 2.2.3.1.1. Visual inspection of nasal inserts

Each insert was tested for the appearance and physical properties including shape, color, and texture.

###### 2.2.3.1.2. Determination of drug content of nasal insert

One insert was dissolved in 100 ml ethanol and the rest of the method as in gel evaluation three times and mean results were taken.

###### 2.2.3.1.3. Uniformity of the weight

The test was carried out according to the European pharmacopeia 2002 for tablet. Twenty inserts, from each formula, were individually weighed. The mean weight of inserts was calculated. The inserts meet the test if not more than two tablets deviate from the average weight by more than 7.5%, and none deviated by more than twice that percentage.

###### 2.2.3.1.4. Friability

Five inserts from each formula were accurately weighed and placed in the drum of a friabilator, rotated at 25 rpm for a period of 4 min. The inserts were then brushed and reweighed. The percentage loss in weights was calculated and taken as a measure of friability.

###### 2.2.3.1.5. Wetting time

Ten milliliters of distilled water containing eosin a water-soluble dye were placed in a Petri dish of 10 cm diameter (Farid et al., 2013). The insert was carefully placed in the center of the Petri dish and the time required for the dye to reach the upper surface of the insert was noted as the wetting time. The test results presented were the average of three determinations.

###### 2.2.3.1.6. Determination of surface pH

Agar solution was prepared by dissolving 2% w/v agar in distilled water by heating under stirring, then poured in to petri dish to solidify at the room temperature (Seager, 1998). The plain inserts were left to swell for 2 h on the surface of agar plate. Surface pH was measured by mean of a pH paper (Whatman full range 1–14) placed on the surface of the swollen inserts. The measurements were performed in triplicate.

###### 2.2.3.1.7. GH release from different prepared nasal insert formulae

Three inserts were used for this test. The mean of results was taken. The method adopted as in case of drug release from gel formulae in 2.2.2.1.6 section.

###### 2.2.3.1.8. Mucoadhesion study of GHSpNGs

As documented in gel evaluation 2.2.2.1.7 section, but use formulated insert instead of 0.5 gm gel.

#### 2.2.4. *In vivo* study of GHSpNGs and GHSpNIs

All experiments were approved by the Institutional Animal Ethics Committee, Cairo University, Egypt and they comply with the ARRIVE guidelines. Fifty-four male Wister albino rats (200–250 gm) were divided in to three groups each contain 18 rats. The different groups were as following: Group 1 received GH solution in distilled water intravenous. Group 2 received GH Spanlastic loaded in nasal gel. Group 3 received GH Spanlastic loaded in freeze dried insert. GH was administered for all animals in a dose of 20 μg/kg. GH solution was injected into the peripheral tail vein of rats. Nasal gel and insert were instilled into nostrils with the help of micro injector equipped with soft polyethylene tube having 0.10 mm internal diameter at the delivery site. At different time intervals 1, 2, 3, 4, 5, and 6 h following administration of GH, rats were scarified. Blood was collected from the trunk in to heparinized tubes centrifuged at 4000 rpm for 15 min and the separated plasma was transferred into tubes. Brain tissue samples were taken after the skulls were cut, opened, and then homogenized with threefold volumes of distilled water at 24,000 rpm for 1 min and transferred into tubes (Fukami et al., 2005; Costantino et al., 2007). Homogenized brain and separated plasma tubes were stored at −80 °C until assayed.

##### 2.2.4.1. Assay of GH in brain and plasma

GH was analyzed in plasma and in the homogenized brain samples using a liquid chromatography tandem mass spectrometry (LC–MS/MS). An aliquot of 20 μl samples was injected into a Shimadzu Prominence (Shimadzu, Japan) series LC system equipped with degasser (DGU-20A3) using Agilent C18 column (50 × 4.6 mm) with 5 μm-particle sizes. The isocratic mobile phase (acetonitrile and 20% water +0.1% formic acid) was delivered at a flow rate of 1.0 ml/min into the mass spectrometer’s electrospray ionization chamber. Quantitation was achieved by MS/MS detection in positive ion mode for both GH and toresemide (IS) using a MDS Scitex (Foster City, CA) API-3200 mass electrometer, equipped with a Turbo interface TM interface at 400 °C. The ion spray voltage was set at 5500 V. The common parameters, *viz.*, nebulizer gas, curtain gas, and collision gas were set at 40, 20, and 3 psi, respectively. The compound parameters, *viz.*, declustering potential (DP), collision energy (CE) entrance potential (EP), and collision exit potential (CXP) were 71, 31, 10, 4 V for GH and 18, 25, 6, 8 V for IS, respectively. Detection of the ions was performed in the multiple reactions monitoring (MRM) mode, monitoring the transition of the *m*/*z* 313.025 precursor ions to the *m*/*z* 138.2 for GH and *m*/*z* 348.988 precursor ions to the *m*/*z* 263.9 for IS. The Q1 and Q3 quadruples were set on unit resolution. The political data were processed by Analyst software (Version 1.5, Foster, CA).

##### 2.2.4.2. Pharmacokinetics analysis

The mean concentrations of GH in plasma and brain samples were plotted against time and the peak plasma and brain concentrations (*C*_max_) as well as the time to reach these peaks were read directly. The area under GH concentration curve (AUC_6 h_) was calculated by the trapezoidal method without extrapolations.

##### 2.2.4.3. Transport study using the *in vivo* rat model

To evaluate brain GH targeting after intranasal dosing, two indices were adopted (Zhang et al., 2004; Costantino et al., 2007).

Drug targeting efficiency (DTE %) that represents time average partitioning ratio was calculated as follows:

$$DTE\%=\left[\frac{\left({\mathrm{AUC}}_{\mathrm{brain}\;\text{0-6h}}/{\mathrm{AUC}}_{\mathrm{blood}\;\text{0-6h}}\right){}_{\mathrm{IN}}}{\left({\mathrm{AUC}}_{\mathrm{brain 0-6h}}/{\mathrm{AUC}}_{\mathrm{blood}\;\text{0-6h}}\right){}_{\mathrm{IV}}}\right]\times 100$$

Nose-to-brain direct transport percentage (DTP %), calculated as follows:

$$DTP\%\;=\;\left[\frac{B{}_{\mathrm{IN}}-B{}_{x}}{B{}_{\mathrm{IN}}}\right]\times 100$$

where B_IN_ is AUC_0–6 h_ (brain) following IV administration, B_x_ is the brain AUC fraction contributed by systemic circulation through the BBB following IN administration, and equals:

$$B{}_{x}=\left[\frac{B{}_{\mathrm{IV}}}{P{}_{\mathrm{IV}}}\right]\times P{}_{\mathrm{IN}}$$

where B_IV_ is AUC_0–6 h_ (brain) following IV administration, P_IV_ is AUC_0–6 h_ (blood) following IV instillation.

## 3. Results and discussion

### 3.1. Differential scanning calorimetry (DSC)

The findings that could point out the possible dispersion of GH throughout the nasal insert and gel in form of spanlastic dispersion with no interaction were referenced Ahmed et al. (2014).

### 3.2. Characterization of GHSpNG formulae

#### 3.2.1. Visual inspection

All GHSpNGs showed homogenous, translucent, and no precipitation.

#### 3.2.2. pH measurement

All GHSpNG formulae exhibited values of pH between 5.38 and 7.01. These values were suitable and non-irritant to the nasal mucosal surface (Osborne & Amann, 1990).

#### 3.2.3. Determination of drug content

Drug content percentages values ranged between 83.38% and 98.6%. It was found that drug content in GHSpNGs decreased with increasing the polymer concentration.

#### 3.2.4. Spreadability of GHSpNGs

The spreadability test is very important as it showed the behavior of gel when it comes out from the tube and during the patient application in the nasal cavity. Spreadability test was carried out for all the formulations. The degree of spreadability of the tested gel formulae was indicated by the distance traveled by the gel when was compressed between the fixed and movable slides. It was found that the spreadability was decreasing with the increase in the concentration of the polymer. The spreadability of GHSpNGs showed in the range (2.35–6.2).

#### 3.2.5. *In vitro* GH release from different prepared GHSpNG formulae

The *in vitro* release of GH from HPMC gels was found faster than carbopol 934 gels. It was observed that all formulae had become liquefied and diluted at the end of the experiment. An inversely proportional relationship between concentrations of each polymer and the percentage of GH released from the gel was observed. When a semi-solid formulation in a unique type of polymer with different concentrations was used, the active substance released from the formulations decreased as the polymer concentration increased. This may be due to that at high polymer concentrations, the active substance was trapped in smaller polymers and was structured by its close proximity to that polymer molecule.

The density of chain structure which had been observed in gels microstructure increased at high polymer concentration and this limited the active substance movement area leading to decrease in the release of the active substance (Wang et al., 2001).

**Figure 1.**
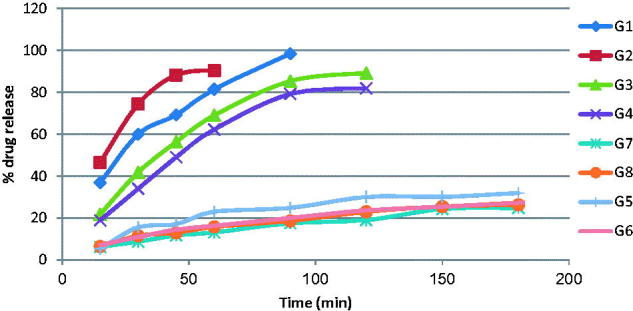
*In vitro* release of drug from the GHSpNGs in distilled water.

The *in vitro* release results of GH from different gel formulae were studied in distilled water. The release profiles are illustrated graphically in Figure 1.

#### 3.2.6. Rheology measurement

All the prepared formulae possessed thixotropic behavior, non-Newtonian shear thinning (pseudoplastic) flow since the viscosity decreased with increasing the shear rate. The reasons of pseudoplastic flow may be due to progressive rupture of the internal structure of the formulations (by increasing shear) and its later reconstruction by means of Brownian movement. By comparing the viscosity values of the gel formulae prepared by the same polymer in different concentration, it was found that by increasing the polymer concentration, there was an increase in the viscosity values in all the formulae (Fang et al., 2003).

#### 3.2.7. Determination of mucoadhesive force

According to the previous results, the chosen two formulae (G1 and G2) with a higher release and good drug content to perform mucoadhesion test. The results of mucoadesive force for the selected formulae (G1 and G2) were 4905 and 5003 dyne/cm^2^, respectively.

### 3.3. Characterization of GHSpNIs formulae

#### 3.3.1. Visual inspection of nasal insert

All formulae had spongy appearance and smooth surface, which were advantageous in placing the insert in the nose with minimum discomfort. All inserts had white color.

#### 3.3.2. Determination of drug content of GHSpNIs

The drug content in the prepared inserts was in the range from 82.4% to 98.38%. This indicates that the adopted method of preparation gave reproducible results and that the drug was uniformly distributed in the polymeric matrix (Dae-Duk, 2007). It was found that the formula of the highest drug content was F8 (98.38%), and the lowest drug content was F6 (82.4%).

#### 3.3.3. Uniformity of the weight

The inserts fall within the acceptable weight variation range (European pharmacopeia limits).

#### 3.3.4. Friability

The inserts did not show any capping or breaking during the test. Results showed that inserts formulated with glycine 1% as a collapsing protectant showed lower percentage of the friability than those with 0.5% glycine.

#### 3.3.5. Wetting time

The average of wetting time of the different insert formulations was in the range 2–15 s. The results revealed that increasing the concentration of collapse protectant, glycine, resulted in longer wetting time. Inserts containing 1% gelatin showed wetting time more than inserts containing 2% HPMC.

#### 3.3.6. pH measurement of the nasal inserts

The prepared inserts pH values were in the range 5.6–6.7. Such pH is very close to the human nasal mucosa to avoid any probable mucosal irritation (Kakkar & Pal Kaur, 2013).

#### 3.3.7. *In vitro* drug release profile from different GHSpNI formulae

**Figure 2.**
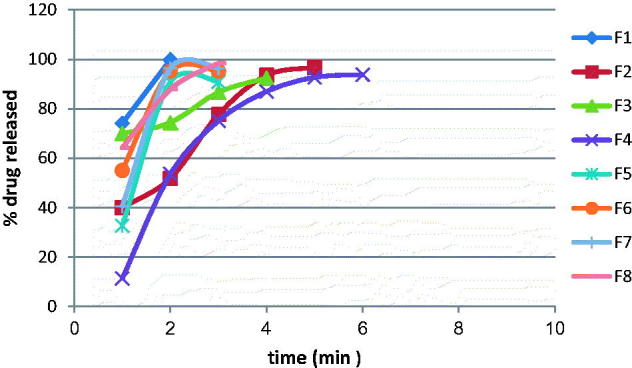
The release of GH from the prepared GHSpNIs.

The cumulative GH release as a function of time from its nasal inserts containing HPMC (2%) or gelatin (1%) and glycine at two different concentrations 0.5% and 1% was studied. Figure 2 shows the cumulative percentage of GH release as a function of time from the nasal inserts. HPMC type nasal inserts containing (1%) glycine (F3, F4) had slower release rate than that containing (0.5%) glycine (F1, F2). On the other hand, gelatin type nasal inserts containing 0.5% glycine (F5, F6) had slower rate than that containing 1% glycine (F7, F8). The nasal insert formula (F1) showed the highest release rate than other formulae.

#### 3.3.8. Mucoadhesion of the nasal inserts

Based on the calculated drug content and the *in vitro* drug release profile, the nasal insert formulae (F1, F8) were selected for the mucoadhesion test. The mucoadhesion force for the selected nasal inserts (F1, F8) were (6867, 5396 dyne/cm^2^), respectively. According to the types of polymer mucoadhesion force of HPMC type nasal insert F1 was greater than the mucoadhesion force of gelatin type nasal insert F8.

### 3.4. *In vivo* pharmacokinetic analysis

**Figure 3.**
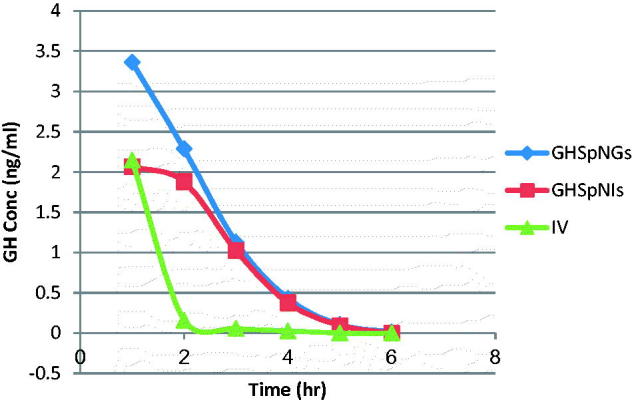
Mean GH concentration–time profile in the plasma of rats for (IV, GHSpNGs, and GHSpNIs).

**Figure 4.**
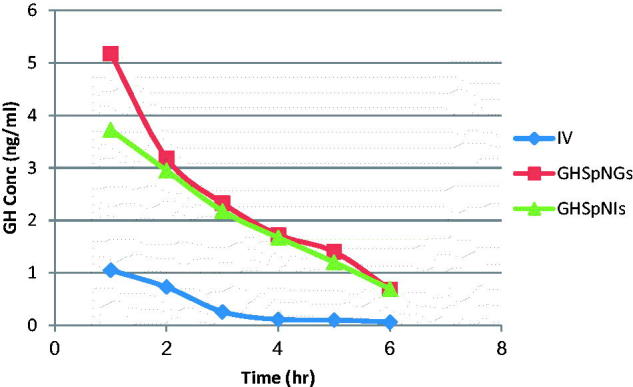
Mean GH concentration–time profile in the brain of rats (IV, GHSpNG, and GHSpNI).

Figure 3 shows the mean GH concentrations in plasma of rats after administration of IV GH solution, GH intranasal gel and insert. Rapid decline of GH concentration after IV administration is observed in Figure 3 but for GH spanlstic loaded in nasal insert and gel showed more resistance due to protection of the drug by spanlastics. The corresponding pharmacokinetic parameters in term of *C*_max_ in plasma were 2.15 ± 0.22, 3.36 ± 0.75, and 2.07 ± 0.07 ng/ml for IV, GHSpNGs, and GHSpNIs, respectively. In term of AUC_0–6 h_ were 0.27 ± 2.38, 7.32 ± 0.98, and 5.4 ± 0.18 h ng/ml for IV, GHSpNGs, and GHSpNIs, respectively. The *C*_max_ of GH in plasma after IN administration of gel and insert were 1.56 times and the same after IV administration of drug solution, respectively. The AUC_0–6 h_ after administration of IN gel and insert were 3 and 2.2 times that after IV administration of drug solution, respectively. The observed increase in the AUC(0–6 h) of GH nasal gel and insert could be attributed to several factors explained as follows: First, the lipophilic nature and elasticity nature of spanlastics being helped the particles to partition into the nasal epithelial cell and pass directly through the cells, thus showing higher systemic absorption (Costantino et al., 2007; Kaur et al., 2008; Kozlovskaya et al., 2014). Second, it is reported that incorporation of specialized reagents (e.g. absorption enhancers, mucoadhesive compounds, etc.) could increase the efficiency of drug delivery to the brain *via* nasal route (Chaturvedi et al., 2011). HPMC used in the nasal gel and insert preparation also contributed to the enhanced nasal absorption. HPMC had a desirable mucoadhesive property and hence prolongs drugs residence time in the nasal cavity so it could act as absorption enhancer, thus increasing the bioavailability (Pardridge, 1998). The mean GH concentrations in brains of rats after administration of IV GH solution, GH spanlastic loaded nasal gel (GHSpNG), and GH spanlastic loaded nasal insert (GHSpNI) are shown in Figure 4. The pharmacokinetic parameters in term of *C*_max_ in brain were 1.05 ± 0.05, 5.17 ± 1.12, 3.72 ± 0.47 ng/ml for IV, GHSpNGs, and GHSpNIs, respectively. In term of AUC_0–6 h_ were 2.31 ± 0.18, 14.16 ± 0.84, and 12.08 ± 1.86 h ng/ml for IV, GHSpNGs, and GHSpNIs, respectively. GH concentrations in brain could be detected for one hour only after IN administration of drug and IV administration. In the brain, the gel and insert formulations had *C*_max_ higher than that of IV drug solution. *C*_max_ of gel and nasal insert were 6.22, and 3.5 times that of the IV drug solution, respectively. The enhanced brain absorption of the nasal formulation might be attributed to the composition of spanlastic system including its lipid nature, elasticity, and its surfactant content. The lipophilic spanlastic can entrap the hydrophilic drug GH helping it to cross the BBB *via* transcellular diffusion in which the general rule was the higher the lipophilicity of a substance, the greater the diffusion into the brain (Kreuter, 2001; Kozlovskaya et al., 2014). Surfactants like tween 80 in spanlastics act as absorption enhancers, thus enhancing the transport across the BBB. It was reported that tween 80 can increase that permeation across BBB by several mechanisms (Kreuter et al., 2002). Moreover, tween 80 could decrease the nanoparticles clearance by the reticuloendothelial system (RES), inhibit the efflux system especially P-glycoprotein (Pgp) (Kreuter et al., 2003, 2004; Alam et al., 2010) and increase the brain uptake *via* the transient opening of the brain endothelial tight junctions (Vyas et al., 2005). Anionic spanlastic showed an increased brain penetration. Drug uptake into brain from the nasal mucosa occurs mainly by different pathways. One is the systemic pathway by which some of drug is absorbed into the systemic circulation and subsequently reaches the brain by the crossing the BBB. The other is the olfactory and the trigeminal neural pathway by which drug directly travels from the nasal cavity to CSF and/or brain tissue that can be concluded that the amount of drug in the brain tissue after intranasal administration is attributed to these two pathways (Zhang et al., 2004; Thorne et al., 2004; Kaur et al., 2008). It has to be noted when interpreting the results obtained, that the olfactory transport of drug will be much more pronounced in rats than in humans (Kaur et al., 2008). This might be attributed to two reasons including: First, the increased surface area of the olfactory region in rat (50%) of the total nasal epithelium compared to that in human (only 3%) (Van Dongen, 1998). Second, the weight of the brain of the rat (2 g) is much smaller than that of the human (1300–1400 g) leading to increased penetration of drugs into the rat brain (Yu et al., 2009).

The values of DTE% and DTP% were 199.3, 49.8% for group 2 and 230, 56.95% for group 3, respectively. The higher the DTE% the greater the degree of drug targeting to the brain that could be expected after intranasal administration (Kaur et al., 2008; Kozlovskaya et al., 2014). DTP% represented the percentage of drug directly transported to the brain *via* the olfactory and trigeminal pathways by subtracting the part contributed by the systemic circulation through the BBB. The DTP% suggested that the main drug transport to brain was via the olfactory and trigeminal route for gel and insert. The higher DTE% and DTP% for the in gel and insert indicated their efficiency in targeting drug to the brain directly through the nose (Zhang et al., 2004; Kumar et al., 2008).

## 4. Conclusion

On the basis of the present study, it could be concluded that GH spanlastics loaded in a nasal gels and inserts are a promising to improve GH bioavailability and provide high drug brain levels.
